# Supplementary material for: Association of the Posterior Acromion Extension with Glenoid Retroversion: A CT Study in Normal and Osteoarthritic Shoulders
Source: J Clin Med. 2022 Jan 12;11(2):351. doi: 10.3390/jcm11020351 (PMC8779855; doi:10.3390/jcm11020351)
Supplement: Supplementary file 1 [file jcm-11-00351-s001.zip › jcm-1480679-supplementary.pdf]

Supplementary Materials

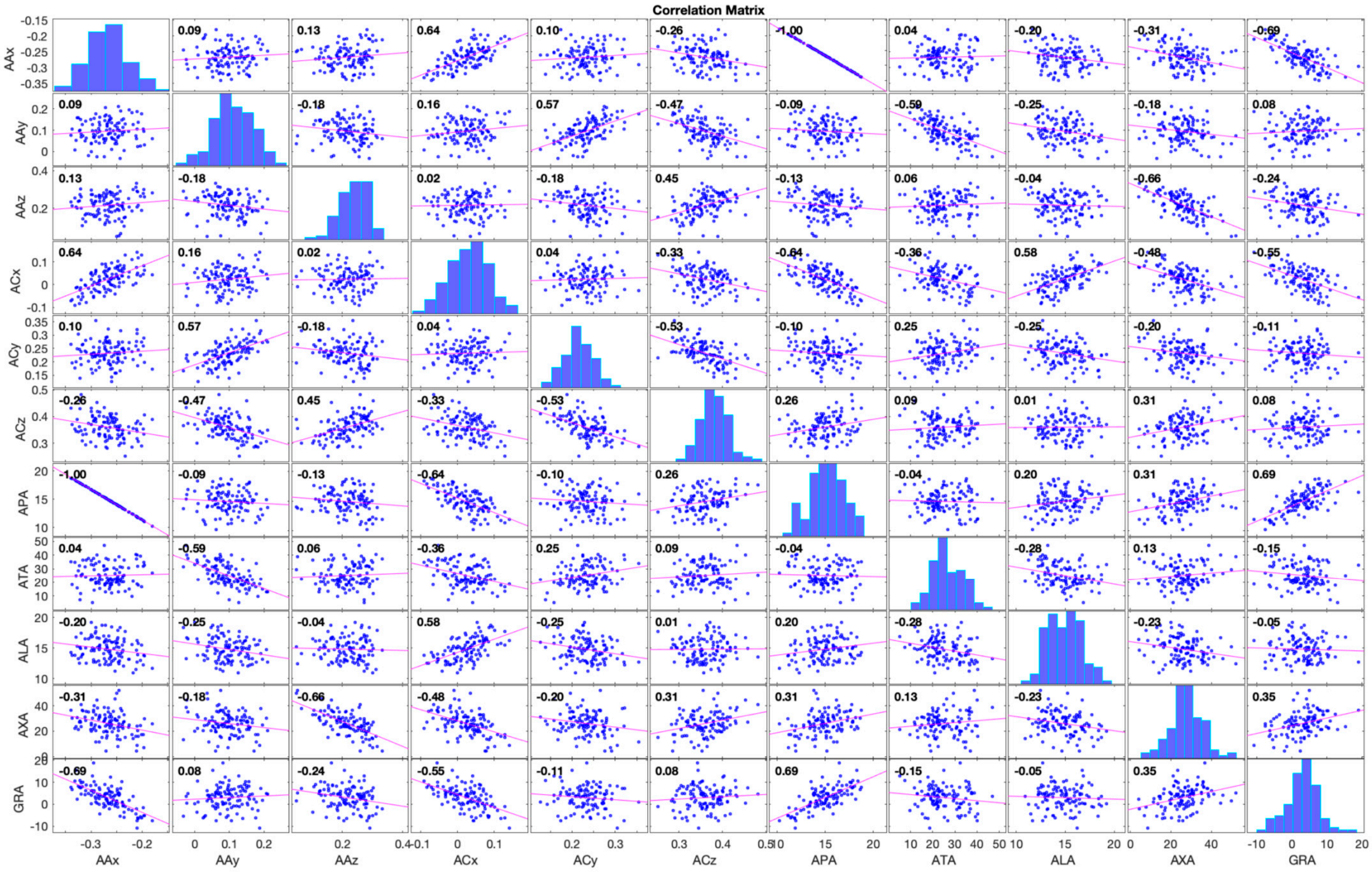

Figure S1. Correlation matrix among acromion landmarks, acromion angles and glenoid retroversion angle.
